# Meals Medicinal: With "Herbal Simples." Curative Foods from the Cook; in Place of Drugs from the Chemist

**Published:** 1905-03

**Authors:** 


**Meals Medicinal: with "Herbal Simples." Curative Foods from the Cook; in place of Drugs from the Chemist.** By W. T. Fernie, M.D. Pp. xxii., 781. Bristol: John Wright & Co. 1905.

The author of *Herbal Simples* writes this larger work for the purpose of instructing readers how to choose meats and drinks which can afford precisely the same remedial elements for effecting cures as medicinal drugs have hitherto been relied on to bring about. The mênu provides a complete dispensatory of remedial diet applicable to the treatment throughout of most diseases and ailments. Its *modus medendi* is made lucid and plain. Many are the digressions which give sunshine and life to the subject; much dexterity has been shown in the management and good cooking of these. The book is dedicated "to our 'Little Marys,' with the homage of a lifetime spent in their service"; it is full of information so well served that it is everywhere appetising and easily digested. "Take therefore, gentle readers, in good part what's projected for thee: so shall our pains not quite want their recompense; nor thyselves be branded with the base mark of ingratitude."

